# Hepatitis B Virus Capsid: The Core in Productive Entry and Covalently Closed Circular DNA Formation

**DOI:** 10.3390/v15030642

**Published:** 2023-02-28

**Authors:** Megan A. Mendenhall, Xupeng Hong, Jianming Hu

**Affiliations:** Department of Microbiology and Immunology, The Pennsylvania State University College of Medicine, Hershey, PA 17033, USA

**Keywords:** hepatitis B virus, cccDNA, capsid, HBc, infection, host factors, host tropism, entry

## Abstract

Hepatitis B virus (HBV) relies on the core protein (HBc) to establish productive infection, as defined by the formation of the covalently closed circularized DNA (cccDNA), as well as to carry out almost every step of the lifecycle following cccDNA formation. Multiple copies of HBc form an icosahedral capsid shell that encapsidates the viral pregenomic RNA (pgRNA) and facilitates the reverse transcription of pgRNA to a relaxed circular DNA (rcDNA) within the capsid. During infection, the complete HBV virion, which contains an outer envelope layer in addition to the internal nucleocapsid containing rcDNA, enters human hepatocytes via endocytosis and traffics through the endosomal compartments and the cytosol to deliver its rcDNA to the nucleus to produce cccDNA. In addition, progeny rcDNA, newly formed in cytoplasmic nucleocapsids, is also delivered to the nucleus in the same cell to form more cccDNA in a process called intracellular cccDNA amplification or recycling. Here, we focus on recent evidence demonstrating differential effects of HBc in affecting cccDNA formation during de novo infection vs. recycling, obtained using HBc mutations and small molecule inhibitors. These results implicate a critical role of HBc in determining HBV trafficking during infection, as well as in nucleocapsid disassembly (uncoating) to release rcDNA, events essential for cccDNA formation. HBc likely functions in these processes via interactions with host factors, which contributes critically to HBV host tropism. A better understanding of the roles of HBc in HBV entry, cccDNA formation, and host species tropism should accelerate ongoing efforts to target HBc and cccDNA for the development of an HBV cure and facilitate the establishment of convenient animal models for both basic research and drug development.

## 1. Introduction

Hepatitis B virus (HBV) belongs to the *Hepadnaviridae* family [1] and is responsible for the infectious liver disease, hepatitis B. According to the World Health Organization’s estimation, about 294 million individuals worldwide are chronically infected with HBV [2], who are at high risk for developing liver cirrhosis and hepatocellular carcinoma. HBV is an enveloped DNA virus [3]. The outer lipid viral envelope contains three surface antigens, large (L), middle (M), and small (S), which cover the viral capsid that is composed of 240 copies (T = 4) or 180 copies (T = 3) of the HBV core protein (HBc). Inside the capsid, a small (ca. 3.2 kbp) genome in the form of relaxed circular DNA (rcDNA), which is partially double-stranded, is covalently attached to the viral DNA polymerase (Pol—a specialized reverse transcriptase (RT)) [4,5]. In addition to the infectious viral particle (i.e., DNA-containing virion or complete virion), infected hepatocytes also produce empty virions, which consist of the surface antigens and the capsid but no packaged viral genome (either RNA or DNA) [6], and low levels of RNA-filled virions [7].

**Figure 1:**
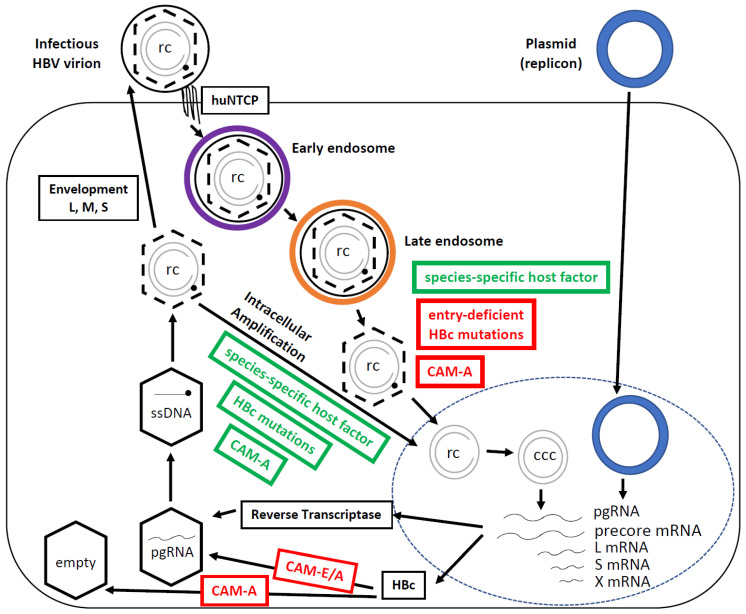
The simplified HBV lifecycle. HBV is endocytosed, traffics through the early (purple) and late (orange) endosomal compartments, undergoes cytosolic release and nuclear import to deliver rcDNA to the nucleus, where it is converted to cccDNA. Transcription and translation then occur, and nucleocapsid packaging of pgRNA and conversion to rcDNA ensues. rcDNA-containing nucleocapsids can be enveloped and secreted or cycled back to the nucleus to make more cccDNA (intracellular amplification). The dashed lines of capsids (hexagons) denote capsid destabilization associated with maturation (rcDNA synthesis), which is thought to prime the mature nucleocapsid for uncoating. The steps impaired (red) or enhanced (green) by HBc mutations and CAMs, or facilitated by species-specific host factors (green), are highlighted. HBV replication steps not directly relevant here are omitted for brevity. Plasmid (HBV replicon) transfection, as depicted, bypasses the entire HBV entry steps to deliver the HBV DNA (cccDNA equivalent) to the nucleus. CAM-A—capsid assembly modulator-aberrant; CAM-E/A—capsid assembly modulator-empty/aberrant; huNTCP—human sodium taurocholate co-transporting polypeptide; ssDNA—single-stranded DNA; rc—rcDNA; ccc—cccDNA.

HBc, a 21 kDa protein translated from HBV pregenomic RNA (pgRNA), is the structural subunit of the viral capsid. HBc contains an N-terminal domain (NTD, residues 1–140) and an arginine-rich C-terminal domain (CTD, residues 150 to 183 or 185, depending on the genotype) that are connected by a short linker peptide (residues 141–149) [5,8]. The NTD is essential for capsid assembly but also facilitates the packaging of pgRNA and the reverse transcription of pgRNA to produce rcDNA, and the CTD is essential for pgRNA packaging and rcDNA synthesis but also controls capsid assembly under physiological conditions [5,9,10,11,12,13,14]. In addition, the arginine-rich sequences on the CTD have nuclear localization signals (NLSs) and nuclear export signals (NESs), which are essential for the bidirectional trafficking of HBc or the capsid [15,16]. The linker region was generally assumed as a mere spacer between NTD and CTD. However, recent evidence indicates that the linker region plays critical roles in multiple steps of HBV replication, including viral entry and cccDNA intracellular amplification, consistent with its high sequence conservation [9,10]. Furthermore, HBc is involved in regulating cytosolic trafficking and the nuclear delivery of rcDNA. Critically, HBc also controls the release of rcDNA from the nucleocapsid in a process called uncoating (nucleocapsid disassembly), which is essential for the formation of the HBV covalently closed circular DNA (cccDNA) in the nucleus, the critical viral DNA species that establishes and sustains HBV infection and persistence [9,17,18]. Thus, HBc plays a critical role in almost all steps in the HBV lifecycle (Figure 1). There have been several excellent reviews on HBc structure and functions recently [4,5,8]. Here, we will highlight the roles of HBc specifically in infectious entry and cccDNA formation.

## 2. The HBV Lifecycle

The HBV lifecycle (Figure 1) begins when the infectious viral particle (i.e., the complete virion) attaches to human hepatocytes via heparan sulfate proteoglycan (HSPG) [19,20], followed by a high-affinity interaction of the PreS1 domain of the HBV large surface protein (L) with the receptor, human sodium taurocholate co-transporting polypeptide (huNTCP), a bile acid transporter [21,22]. Following receptor binding, most evidence suggests that HBV is internalized via clathrin-mediated endocytosis [23,24,25]. The epidermal growth factor receptor (EGFR) is a co-factor involved in the huNTCP-medicated internalization of HBV [26]. HBV then traffics through the early and late endosomes [27,28]. HBV infection is likely pH-independent [29,30,31,32,33]. Current evidence suggests that the virion-derived nucleocapsid escapes from the late endosome into the cytosol following virus–cell membrane fusion [28,34], which is thought to be mediated by both the PreS1 domain of L and the small surface protein (S) [34,35,36,37]. A recent study proposed that S participates in a thiol–disulfide exchange reaction that leads to the exposure of the PreS1 region of L, which allows for membrane fusion [34]. Interestingly, recent cryo-EM structural studies of HBV surface antigen (HBsAg) subviral particles, composed of mostly S, have indicated that there is no canonical lipid bilayer on these particles [38]. Assuming HBV virions similarly lack a lipid bilayer, HBV may use a mechanism for delivering the nucleocapsid into the cytosol that is rather different from classical virus–cell membrane fusion. Upon release of the nucleocapsid from the endosomal compartment, it is thought to traffic through the cytosol and then deliver rcDNA into the nucleus, where rcDNA is converted to cccDNA via host DNA repair mechanisms [39]. As cccDNA is the first new viral product generated during infection and essential for subsequent viral gene expression, cccDNA formation during infection is considered the hallmark of productive or infectious entry.

Structurally, cccDNA forms a mini-chromosome with host cell histones and acts as the template for viral RNA transcription by the host RNA polymerase II [40]. pgRNA, in addition to serving as the mRNA for HBc (see above) and RT, also serves as the template for reverse transcription to produce progeny viral DNA. In the cytoplasm, RT binds pgRNA, and both, together, are encapsidated into an immature nucleocapsid formed by HBc. Progeny rcDNA is then produced from pgRNA by RT inside the nucleocapsid via a single-stranded DNA (ssDNA) intermediate. Like pgRNA-containing nucleocapsids, ssDNA-containing nucleocapsids are also considered immature as both are incompetent for secretion as virions. In contrast, rcDNA-containing (i.e., mature) nucleocapsids can be enveloped by the viral surface proteins and secreted as complete virions [41]. Alternatively, mature nucleocapsids newly formed in the cytoplasm can deliver the progeny rcDNA back into the nucleus to replenish the nuclear cccDNA pool in a process called intracellular cccDNA amplification or recycling [42,43,44]. As HBc (capsid) is a critical player in almost every step of the HBV lifecycle, it has emerged as an attractive antiviral target [4].

## 3. Role of HBc in Viral Entry—Cytoplasmic Trafficking, Uncoating, and Nuclear Import

Although huNTCP has been identified as a bona fide HBV entry receptor for a decade [21], the entry process of HBV remains poorly understood. Upon release of the virion nucleocapsid into the cytosol, several lines of evidence suggest that the microtubule-dependent transport is responsible for its trafficking through the cytoplasm [45,46]. It was reported via confocal laser scan microscopy that capsids distribute throughout the cytoplasm and accumulate at the nuclear envelope [45]. Furthermore, with the inhibition of microtubules using the microtubule-destabilizing inhibitor nocodazole, cccDNA was not produced after mature (rcDNA-containing) nucleocapsids were introduced into the cytoplasm by transfection [45]. A more recent study indicated that HBV capsids interact with tubulin directly, and co-localize with microtubule-like fibriforms [47]. Nucleocapsids are also reported to interact with the dynein motor complex and the dynein light chain for trafficking through the cytoplasm to the nuclear pore [46]. Once the nucleocapsid reaches the nuclear pore, it is thought to deliver rcDNA to the nucleus. The nuclear pore complex (NPC) transports particles up to 39 nm in diameter or even larger [48]. Whether the nucleocapsid, with a diameter of 36 nm, partially or fully disassembles to enter the nucleus is unclear. The HBc NLSs overlap with the CTD arginine-rich sequences [15,49]. It was reported that the phosphorylation of HBc is required for NLS exposure [50,51]. The HBc NLSs interact with importin alpha/beta and also with nucleoporin 153 [52,53], which may facilitate nucleocapsid disassembly for delivery of rcDNA into the nucleus. Although nucleocapsid disassembly (uncoating) and the nuclear import of rcDNA are two obvious prerequisites for cccDNA formation, these processes remain incompletely understood. It is clear, however, that at least a portion of mature nucleocapsids is structurally destabilized, as compared to immature ones, becoming porous and allowing local exposure of the rcDNA content [54,55]. These results suggest that mature nucleocapsids are “primed” for uncoating, and rcDNA may be released from mature nucleocapsids without their complete disassembly [54,55].

## 4. Role of HBc in cccDNA Formation during Intracellular Amplification

Both intracellular amplification and de novo infection pathways are responsible for the maintenance of the cccDNA pool in HBV-infected hepatocytes [3,56] (Figure 1). The transfection of HBV DNA replicon into cells that support intracellular HBV replication events but not the viral entry steps provides an approach to study cccDNA formation via intracellular amplification in the absence of de novo infection. Studies using this transfection system have uncovered a critical role of HBc in cccDNA formation independent of its roles in viral entry. In principle, HBc can regulate cccDNA formation via at least three distinct mechanisms. Firstly, HBc can negatively regulate cccDNA formation via nucleocapsid envelopment and virion secretion by interacting with the viral envelope proteins. This phenomenon has been demonstrated via mutations in either HBc or the L surface protein that impair nucleocapsid envelopment and virion secretion and increase cccDNA formation via intracellular amplification [9,44,57,58,59]. Secondly, HBc can regulate cccDNA formation by controlling the disassembly of mature nucleocapsids (uncoating). This process is known to be regulated by dynamic HBc phosphorylation and dephosphorylation [9,18,60]. In addition, HBc NTD or linker mutations that selectively impair the integrity/stability of mature nucleocapsids, such as I126A and S141A, show enhanced cccDNA formation [9,58], likely by enhancing uncoating and the release of rcDNA, which may or may not involve alterations in the HBc phosphorylation state. Thirdly, HBc can regulate the nuclear import of rcDNA [11,51]. HBc mutations such as L95A and K96A enhance intracellular cccDNA amplification in a manner that is independent of surface protein interaction and without impacting nucleocapsid uncoating. These mutants are, thus, suspected to enhance the nuclear import of rcDNA although this has not been demonstrated directly [58,61]. In addition, mutations of two putative HBc NTD phosphorylation sites (S44 and S49) led to altered ratios of cccDNA to the closed minus rcDNA (cM-rcDNA), which contains a covalently closed minus strand with the polymerase removed but still an open plus strand and is a likely intermediate in cccDNA synthesis [60]. These altered ratios suggest that HBc may directly modulate the biochemical steps during cccDNA synthesis in the nucleus.

## 5. HBc Mutations That Differentially Affect the Intracellular Amplification and De Novo Infection Pathways

**Figure 2:**
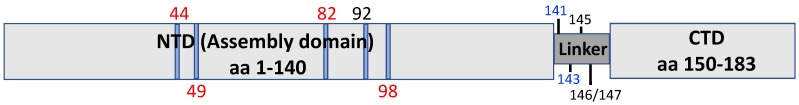
Schematic diagram of HBc domains highlighting residues important for viral entry. HBc residues, in the NTD and linker, shown to play a role in HBV entry are highlighted. Mutations at these positions can block cccDNA formation during HBV infection but allow/enhance cccDNA intracellular amplification. Mutations highlighted in red likely affect cccDNA formation via altered HBc phosphorylation state. Mutations marked in blue affect cccDNA formation via destabilization of nucleocapsids. Mutations listed in black affect cccDNA formation via an unknown mechanism, possibly through cytosolic transport or nuclear import. See text for details.

One of the most intriguing observations regarding the role of HBc in infection and cccDNA formation is that HBc mutations (and inhibitors, see below) can differentially affect cccDNA formation during de novo infection vs. intracellular amplification. Nucleocapsid uncoating, rcDNA nuclear import, and DNA repair are all common steps shared by both incoming nucleocapsids released from virions during infection and progeny nucleocapsids formed in the cytoplasm during intracellular amplification. A number of HBc mutations scattered throughout the NTD (at S44, S49, R82, N92, and R98) and the linker region (at S141, L143, E145, T146, and T147) were recently found to allow, or even enhance, cccDNA formation via intracellular amplification but to impair cccDNA formation during infection (Figure 2) [9,18,59,60]. This selective defect in cccDNA formation during entry but not intracellular amplification, thus, implicates a specific role of the HBc NTD and linker during productive HBV entry (Figure 1). This further suggests that the nucleocapsid released from the incoming virion is somehow differentiated from the progeny nucleocapsid produced in the cytoplasm during the processes leading to cccDNA formation. In agreement with this notion, cytoplasmic progeny nucleocapsids and incoming virion-derived nucleocapsids were shown to have different sensitivity to a cytosolically expressed nuclease, which also led to the suggestion that the disassembly of virion-derived nucleocapsids and progeny nucleocapsids, newly formed in the cytoplasm, may occur at different subcellular compartments [62].

Three potential mechanisms might be responsible for the failure in cccDNA formation of HBc mutants selectively during infection but not during intracellular amplification. First, the hyper-destabilization of mutant HBV nucleocapsids (e.g., S141A and L143I) may be involved. For example, during the passage of the virion through the endosomal pathway, the nucleocapsid within the virion may be altered by the endosomal environment. Therefore, the hyper-destabilized mutant nucleocapsids released from the virions become incompetent to support HBV cccDNA formation via de novo infection, e.g., being unable to complete their journey through the cytosol towards the nucleus or becoming prematurely disrupted/degraded, despite their ability to support or even increase cccDNA formation via the intracellular amplification pathway, whereby mature nucleocapsids formed in the cytoplasm, without ever going through the endosome system, are involved [9,58]. Second, some HBc mutants might have defects in the re-phosphorylation of dephosphorylated mature HBV nucleocapsids released from virions during entry, which normally facilitates uncoating. HBc S44 and S49 are two putative phosphorylation sites in the NTD, which are potential targets of CDK2 (Cyclin-Dependent Kinase 2, a host kinase involved in phosphorylation of the serine–proline motif) [60], and R82 and R98 are part of the consensus cyclin-docking motifs (RXL) that are involved in recruiting CDK2 to be packaged into capsids and to phosphorylate HBc [18]. Therefore, HBc mutants S44A/S49A, R82A, and R98A likely block nucleocapsid uncoating due to the impaired re-phosphorylation of mature nucleocapsids during de novo infection. On the other hand, CDK2 packaging into capsids and the re-phosphorylation of HBc by CDK2 do not appear to be as critical for cccDNA formation during intracellular amplification, possibly due to accessibility to alternative kinases. Unlike the dynamic HBc phosphorylation that plays a pivotal role in uncoating, the two conserved lysine residues on HBc (K7 and K96), and by extension, any potential lysine-specific post-translational modifications (PTMs) on these two lysine residues are dispensable for nucleocapsid uncoating and cccDNA formation [61]. Furthermore, HBc K7 and any potential PTMs on this lysine residue are not required for the HBV entry process, either [61]. Third, the HBc N92T mutant, which shows no apparent hyper-destabilization and is not expected to alter CDK2 packaging or CDK2-mediated phosphorylation of HBc [59], may suggest yet another mechanism. As nucleocapsid disassembly (uncoating), the nuclear import of rcDNA, and the actual DNA repair reactions are presumably shared between cccDNA formation via intracellular amplification and de novo infection, an upstream step(s) before these processes, for example, cytosolic transport of the nucleocapsid released from the virion, is involved in the failure in cccDNA formation of HBc N92T, specifically during infection but not during intracellular amplification. On the other hand, it has been reported that different DNA repair/processing factors may be involved in cccDNA formation during infection vs. intracellular amplification [63], suggesting that some event(s) during nuclear import or even within the nucleus may also contribute to the differential effects of HBc N92T (and possible the other HBc mutants described above) on cccDNA formation via these two different pathways. Future studies will be needed to further test these and, possibly, other mechanisms of how HBc mutations (and also inhibitors, see below) exert these interesting differential effects on the two different pathways of cccDNA formation.

## 6. HBc Inhibitors Differentially Affect the Two Different Pathways of cccDNA Formation

Similar to the HBc mutations discussed above, a number of small molecules, so-called core inhibitors (CIs) or capsid assembly modulators (CAMs), have been identified that exert similar differential effects on cccDNA formation, inhibiting cccDNA formation during infection but permitting/enhancing cccDNA formation via intracellular amplification (Figure 1) [4,64]. CAMs are currently classified into two groups based on their effects on capsid assembly by HBc [65]. Some CAMs induce HBc to form aberrant polymers (CAM-A or aberrant), thus preventing pgRNA packaging as well as the formation of empty HBV capsids, which are normally formed at high efficiency in human cells without packaging any RNA or DNA (Figure 1) [6,66]. Other CAMs allow the formation of empty capsids but prevent the packaging of pgRNA (CAM-E or empty). Interestingly, several CAMs, mostly within the CAM-A group, can also inhibit cccDNA formation during infection by targeting the virion-derived nucleocapsid (Figure 1) [4,64,67,68]. Some of these CAMs were shown to hyper-destabilize, selectively, the mature nucleocapsid, akin to the hyper-destabilizing HBc mutations discussed above. Furthermore, they also allow or even enhance cccDNA formation via intracellular amplification despite their inhibition of cccDNA formation during infection, exactly like the HBc mutants described above and, presumably, via a similar mechanism(s) [64]. Indeed, one such CAM-A, BAY41-4106, was shown to direct HBc to the autophagy system [69], supporting the notion that altered cytoplasmic trafficking of the virion-released nucleocapsid, induced by some CAMs or HBc mutations, may be responsible for the selective block to cccDNA formation during infection but not intracellular amplification.

## 7. The Role of HBc in Specifying HBV Host Tropism

HBV infections are limited to hepatocytes of humans and chimpanzees [70]. The discovery of the HBV entry receptor huNTCP, specifically expressed on human hepatocytes, allowed the identification of one major host-species- and tissue-specific determinant of HBV infection [21,71,72,73,74]. Indeed, the ectopic expression of huNTCP in primary pig and macaque hepatocytes in culture renders them susceptible to HBV and hepatitis D virus (HDV) (a satellite virus of HBV that shares the envelope/surface proteins with HBV) [73,74]. However, mouse, rat, and dog hepatocytes with the ectopic expression of huNTCP can support HDV infection but remain refractory to HBV infection [74]. Similarly, huNTCP-transgenic mice could support HDV infection but remain non-susceptible to HBV infection [75]. Because HBV and HDV share the same early entry events (at least cell attachment and endocytosis), these results implicate species-specific host factors that function following these early entry steps in determining HBV species tropism (Figure 1).

Mouse hepatocytes are also unable to support HBV cccDNA formation via intracellular amplification despite their ability to support efficient HBV gene expression, nucleocapsid assembly, rcDNA synthesis, and virion secretion (Figure 1) [76]. Unlike normal mouse hepatocytes and other mouse hepatic cell lines [77], one immortalized mouse hepatocyte cell line, AML12, was shown to support efficient cccDNA formation via intracellular amplification [17]. Moreover, the ectopic expression of huNTCP in AML12 cells rendered them susceptible to HBV infection, although the infection efficiency remained much lower than human hepatoma cells expressing huNTCP [71] (see below also). Furthermore, the ability to support HBV cccDNA formation in AML12 cells could be correlated with enhanced destabilization (uncoating) of mature HBV nucleocapsids [17]. Similarly, some evidence has been presented recently suggesting that the failure to support nucleocapsid uncoating by mouse hepatocytes may also contribute to the inability of mouse hepatocytes to support cccDNA formation during HBV infection after huNTCP expression [78]. Consistent with the expectation that the DNA repair reactions involved in rcDNA to cccDNA conversion, per se, are highly conserved during evolution, mouse, and even yeast, DNA repair factors can support cccDNA formation from rcDNA mimics [78,79,80]. Therefore, overcoming the failure of nucleocapsid disassembly in mouse hepatocytes should help to establish a mouse model that can support HBV cccDNA formation and infection.

The infection of woodchucks by the woodchuck hepatitis virus (WHV), which is closely related to HBV, has been used widely as an in vivo model system to study HBV infection in humans. Due to the strict species specificity, HBV is unable to infect woodchucks. On the other hand, two woodchuck hepatic cell lines were recently shown to support all intracellular steps of HBV replication, including cccDNA formation via intracellular amplification [72]. Upon huNTCP expression, both cell lines can also support HDV infection. Interestingly, only one (WCH-17), but not the other (WC3), woodchuck hepatic cell line can support HBV infection, as evidenced by cccDNA formation from the incoming virions [72]. These results suggest that a late entry step(s), after huNTCP binding and internalization that are shared with HDV infection, but prior to the nuclear import of rcDNA, determines the differential ability to support HBV cccDNA formation during infection between these two woodchuck cell lines (Figure 1). Similarly, although the mouse AML12 cells can support robust HBV cccDNA formation via intracellular amplification, they can only support low levels of cccDNA formation via de novo infection [71], again implicating a (partial) late entry block to HBV infection in these mouse hepatocytes. Thus, overcoming this putative late entry block, in addition to the uncoating block discussed above, would be necessary to establish an efficient HBV-susceptible mouse model.

Intriguingly, the putative late entry block to HBV infection in non-susceptible cells by WT HBV may be the same or similar to that encountered, in susceptible cells, by the HBc mutants described above as they are also competent to support cccDNA formation during intracellular amplification but not infection (Figure 1). These results suggest that HBc may interact with a host factor(s) to facilitate this late entry step during infection, which plays a critical role in HBV species tropism. On the other hand, the putative host susceptibility factor operating at this late entry stage does not appear to be hepatocyte-specific, as the non-hepatic human embryonic kidney cells (HEK293), upon huNTCP expression, can support cccDNA formation [72]. This result, together with the fact that HEK293 cells can also support cccDNA formation via intracellular amplification [11,81], indicates that hepatocyte-specific factors are also not essential for nucleocapsid uncoating. Of course, non-hepatic cells, even after huNTCP expression, will remain unable to support the full HBV lifecycle as HBV gene expression cannot take place due to the lack of hepatocyte-specific transcription factors, which are necessary for cccDNA transcription and serve as additional host determinants of hepatocyte tropism of HBV [71,82,83]. For example, the NIH 3T3 fibroblasts were unable to support viral replication (pgRNA synthesis or rcDNA production) unless the hepatocyte-specific (enriched) transcription factors HNF4, RXRα, and PPARα were exogenously supplied, demonstrating that HBV replication is dependent on liver-specific transcription factors [83].

## 8. Perspectives

HBc is the building block of the HBV capsid and plays multiple critical roles in the viral lifecycle. Although the roles of HBc in capsid assembly, pgRNA packaging, reverse transcription, and envelopment are understood relatively well, little is currently understood about the HBV entry steps and nucleocapsid disassembly (uncoating), prerequisites for cccDNA formation and productive infection, or about the exact roles of HBc in these processes. Mounting evidence indicates that the HBV capsid plays an essential role during HBV entry by directing proper cytoplasmic trafficking of the nucleocapsid released from the virion and regulating nucleocapsid disassembly (uncoating), both temporally and spatially, for rcDNA release into the nucleus. The proper control of uncoating is similarly critical for cccDNA formation via intracellular amplification. For both cytoplasmic trafficking and uncoating of nucleocapsids, HBc likely exerts its control via interactions with species- but not hepatocyte-specific host factors, which are likely important determinants of HBV species tropism. Moreover, a number of HBc-targeted compounds have been identified that can modulate HBV entry and nucleocapsid uncoating. A better understanding of these processes should help develop more effective HBc-targeted antivirals to block HBV infection and clear cccDNA, as well as more convenient animal models to facilitate basic HBV research and drug development targeting HBc and other viral and host factors. Progress towards these goals will be accelerated by the recent emergency, and further development, of experimental tools including HBc mutants and pharmacological inhibitors with defined phenotypes/effects on the different steps leading to cccDNA formation and host model systems with well-defined deficiencies in these steps.
